# The Antipsychotic Drug Haldol Modulates IQGAP1-Signaling and Inhibits Cell Proliferation in Triple Negative Breast Cancer Cell Lines

**DOI:** 10.17912/micropub.biology.000823

**Published:** 2023-05-05

**Authors:** Varun J. Iyer, Mahasin A. Osman

**Affiliations:** 1 Department of Medicine, Division of Oncology, University of Toledo Medical Center, Toledo, Ohio, 43614 United States

## Abstract

The signaling scaffold oncoprotein IQGAP1 was identified as a classification and therapeutic biomarker in triple negative breast cancer (TNBC) cell lines. Here, we report that the antipsychotic drug Haldol induces novel protein-protein interactions with IQGAP1 and inhibits cell proliferation in TNBC cell lines. The identified proteins share known functions of IQGAP1 in secretion, transcription and apoptosis and provide further classification tools and potential precision therapeutic targets for Haldol in TNBC.

**Figure 1. Haldol inhibits cell proliferation and differentially promotes novel IQGAP1 interactions in triple-negative breast cancer (TNBC) cell lines.**
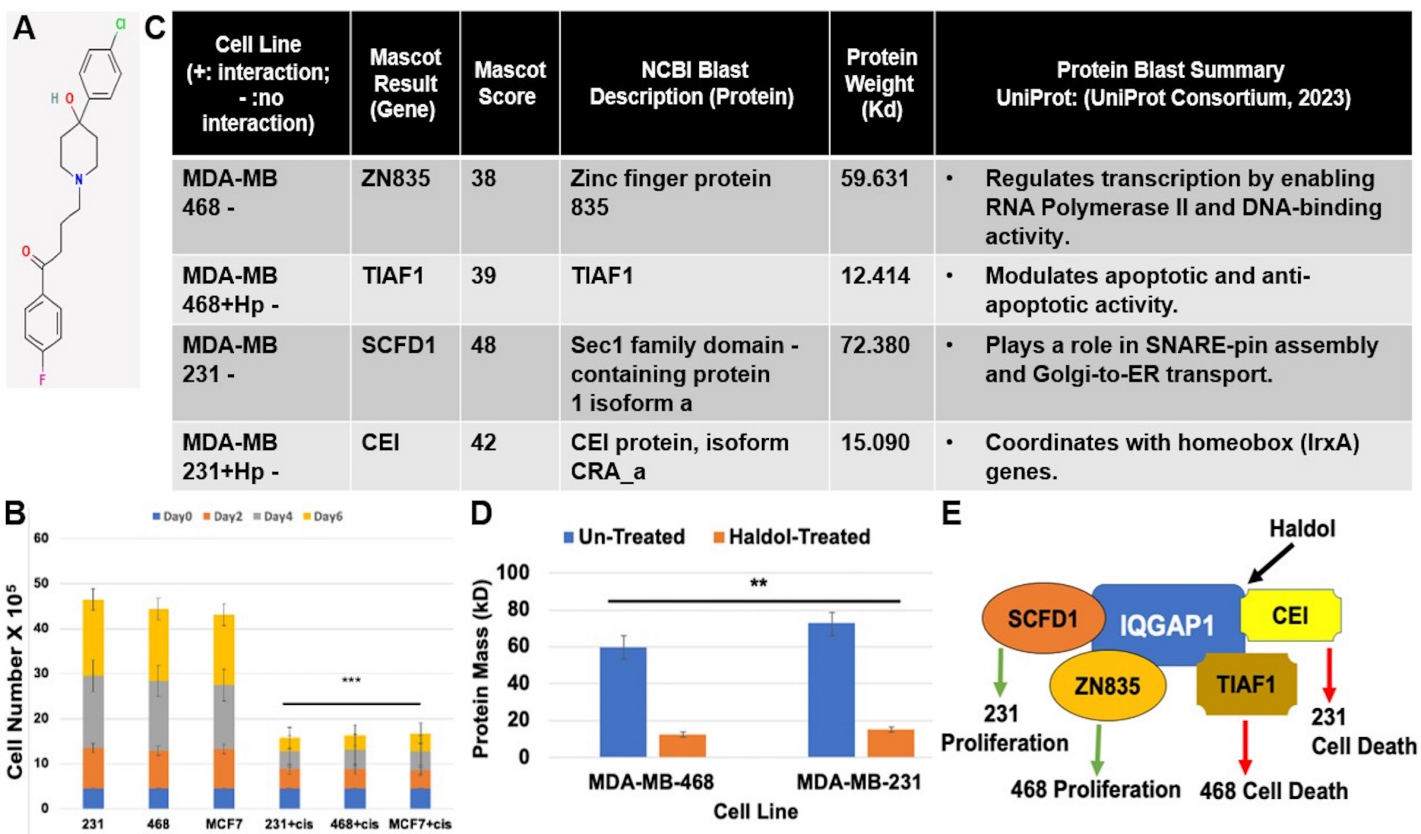
**(A)** Molecular structure of Haloperidol (Haldol, NCBI). **(B)** Haldol (20 μ *M* ) inhibits cell proliferation in the three triple-negative breast cancer cell lines, MDA-MB-231, MDA-MB-468, and MCF7. Error bars are the Means ± s.d for *n = 3* , *** *p =* 0.001 for each of the Haldol-treated cell lines versus their untreated counterparts **(C)** Protein partner identification in IQGAP1-specific immune complexes by mass spectrometry, using MALDI-TOF followed by analyses with the Mascot and NCBI Blast software in control and Haldol-treated TNBC cell samples. **(D)** Protein Mass (kD) comparisons in control and Haldol-treated TNBC cell lines. Error bars are the Means ± s.d for *n = 3* , *p =* ** 0.015 for treated cell lines versus their corresponding controls **(E)** Model of Haldol action on IQGAP1 pathway in TNBC cell lines. The two tested TNBC cell lines are morphologically and molecularly different (Osman et al., 2020). Haldol elicits a different IQGAP1 interaction in each condition, suggesting the activation of distinct pathways leading to cell proliferation inhibition specific for each cell line.

## Description

The triple negative breast cancer is a heterogenous disease defined by the absence of the hormonal and growth factor receptors, thus lacking clinical targets (Foulkes et al., 2010). Recently the oncoprotein IQ-containing Ras GTPase like Protein 1 (IQGAP1) was identified as a possible clinical target and a classification biomarker in TNBC (Osman et al., 2020) . Dysregulation of IQGAP1 shuttling between the centrosome and the nucleus was found to define morphological and molecular differences between the MDA-MB-468 and the MDA-MB-231 TNBC cell lines (Osman et al., 2020) . Normally, IQGAP1 serves as a signaling scaffold hub that nucleates a variety of pathways leading to cell proliferation (White et al., 2012; Osman et al., 2013; Hedman et al., 2015; Osman, 2015) . As such it localizes to various subcellular compartments including the plasma membrane, the centrosome and the nucleus whereby phosphorylation-cycling of IQGAP1 is important for its subcellular localization and role in cytokinesis (Wang et al., 2009; Tekletsadik et al., 2012; Osman et al., 2020) . Additionally, IQGAP1 executes the variety of its cellular functions via differential protein-protein interactions through its various domains associating with a number of signaling and structural molecules, including transcription factors, receptors, kinases, and GTPases (White et al., 2012; Osman et al., 2013; Hedman et al., 2015; Osman, 2015) .

The aim of this study was to further understand the mechanism of IQGAP1 in TNBC by using low-dose small molecule inhibitors as genetic tools in order to define early molecular events leading to growth inhibition. Here, we find that the FDA-approved antipsychotic drug Haloperidol (Haldol) inhibited cell proliferation in several TNBC cell lines, including the MDA-MB 231, MDA-MB-468, and MCF7 at an average of 20 μ *M* ( Figure 1B ). Haldol was reported to inhibit MCF7 cells over-expressing the ABCG2 transporter breast cancer resistance protein (BCRP) at > 100 μ *M* (Wang et al, 2008) , while a Haldol analog, SYA013, was reported to halt cell proliferation in the MDA-MB-231 cell lines at 2-10 μ *M* (Asong et al, 2020) . However, the cellular target and mechanisms of action of Haldol or it’s analogs in cancer cell inhibition remain unknown. Identification of such target(s) and mechanisms is crucial to devising more effective analogs and dosing while avoiding potential adverse side effects of Haldol such as dyskinesia or those that may adversely affect women with established breast cancer (Rahman et al, 2014) . The identification of IQGAP1 and its new partners as plausible clinical targets of Haldol offer potential solution for this problem

To investigate how Haldol might affect IQGAP1 signaling in TNBC, we compared mass spectra of IQGAP1-specific immune complexes to IgG controls obtained from the MDA-MB-231 and MDA-MB-468 TNBC cell lines that were untreated or treated with 20 μm *M* Haldol for 48 hrs. The results revealed novel proteins whose functions are consistent with the known functions of IQGAP1 ( Figure 1C ). Haldol treatment produced a different set of partners in each cell line, suggesting that it modulates IQGAP1 signaling. Furthermore, there was a variation according to the TNBC cell type as each cell line displayed a different molecular signature, consistent with previous findings that these two TNBC cell lines are molecularly different (Osman et al., 2020) .

In the untreated MDA-MB-468 cells, the Zinc Finger DNA-binding protein (ZN835) was isolated as an IQGAP1-binding partner ( Figure 1C ). Zinc finger proteins play a direct role in the binding of transcription factors to DNA gene promoters and are also involved in the regulation of RNA Polymerase II (Laity et al., 2001) . This function is supported by the presence of various DNA binding motifs such as the C _2_ H _2_ domain, leading to triggering transcription as well as controlling transcriptional rates (Filion et al., 2006; Fedotova et al., 2017; Li et al., 2022) . Similarly, IQGAP1 has been shown to alter transcriptional rates via modulating the action of transcriptional co-activators like the Yes-associated protein (YAP) where it binds the N-terminal domain of YAP via its IQ domain (Sayedyahossein et al., 2016) . Furthermore, previous research showed that zinc finger motifs assist in YAP binding to homeoboxes (Lehmann et al., 2016) . Thus, both IQGAP1 and ZN835 modulate transcription by directly binding to transcription factors. More research is underway to reveal what pathway IQGAP1-ZN835 influence; although we can surmise that it has to do with promoting cell survival or proliferation, at least, in the MDA-MB-468 cell lines.

Interestingly, in the untreated MDA-MB-231 cells, the protein traffic related Sec1 family domain-containing protein 1 (SCFD1) was identified ( Figure 1C ). SCFD1 affects the Golgi complex to endoplasmic reticulum (ER) vesicular transport (Hou et al., 2017) . SCFD1 forms pre-Golgi intermediates with the membrane-bound protein Syntaxin to facilitate exocytosis (Halachmi & Lev, 2002; Hou et al., 2017) . Similarly, IQAGP1 was shown to co-localize with Syntaxin, interacts with the exocytosis machinery like Sec3 and Sec6/8 complex and regulate insulin secretion (Osman et al, 2002, Rittmeyer et al, 2008) . Thus, SCFD-IQGAP1 interaction further supports the role of IQGAP1 in exocytosis and protein trafficking. While their exact function in this context requires further investigation, they may synergize to support the metabolic demand of these cancer cells and thus can present a plausible therapeutic target.

In contrast, treatment with Haldol revealed different IQGAP1 interactions in TNBC cell lines, all of which are related to transcriptional activation ( Figure 1C ). In the MDA-MB-468 cells, the relatively understudied TGF- *β* 1-induced antiapoptotic factor (TIAF1) was identified. While TIAF1 was implicated in antiapoptotic activities, a study reported that aggregation of TIAF1 triggers apoptosis in cancer cells (Lee et al., 2010) . Similarly, IQGAP1-BRCA1 aggregates were observed in these cell lines and were suggested to associate with ER stress in cancer (Osman et al., 2020) . It is likely that Haldol promotes IQGAP1-TIAF1 aggregates analogous to the observed IQGAP1-BRCA1 aggregates in these cells (Osman et al., 2020) and instead leads to cell death ( Figure 1B ).

In the MDA-MB-231, Haldol promoted an IQGAP1-Coordinated expression to IRXA2 (CEI) association ( Figure 1C ). The CEI is another understudied protein involved in transcription by an unknown mechanism (Wu et al., 2006) . Sparse research highlighted that CEI interacts with Iroquois genes (IRX), a collection of homeobox genes, in a bi-directional coordinated pipeline to modulate transcription factors (Peters et al., 2000; Wu et al., 2006) . IQGAP1 also coordinates with proteins like Cdc42 in a bi-directional manner to modulate cell morphology and migration (Mataraza et al., 2007) . While further investigation is needed, we can surmise that the directionality of the IQGAP1-CEI association has implications on cell death upon Haldol treatment. Reagents such as ELISA kits for these transcriptional activator proteins have been generated by biotech companies and will facilitate our further investigations of the functions of these novel proteins

The results also highlighted the influence of Haldol on cell mass ( Figure 1D ). This was gleaned from comparing the identified protein mass in treated versus untreated TNBC cells, where protein weight was significantly higher in the untreated cells ( *p=* 0.015, F = 64.360) compared to their treated counterparts. These data are consistent with the observed inhibition of cell proliferation by Haldol ( Figure 1B ) as well as with recent reports that Haldol induces apoptosis in other cell lines via caspase-8 activation (Papadopoulos et al., 2020) .

Overall, using Haldol as a pharmacologic agent, we identified new IQGAP1 partners in a context-dependent manner that highlighted its under-studied role in transcriptional co-activation and that Haldol can modulate IQGAP1 signaling ( Figure 1E ). These partners are potentially useful as clinical biomarkers in TNBC diagnosis and treatment. The data also present the potential for repurposing Haldol or its analogs in treating several types of human cancers in a personalized manner in precision medicine.

## Methods

**Cell Culture:**

The TNBC MDA-MB-468, MDA-MB-231 and MCF7 cell lines were purchased from ATCC and grown in DMEM media supplemented with 1% penicillin-streptomycin and 10% fetal bovine serum (FBS) per ATCC instructions. The cells were grown at 37°C in a sterile humidified incubator supplied with 5% CO _2_ and used at log phase (~ 80% confluency). Cells were discarded after 5 passages and replaced by earlier generations from frozen stocks.

**Cell Proliferation Assay:**

The classic saturation density assay (proliferation rate) was used to measure cell proliferation capacity as previously (Wang et al., 2009) . Briefly, glioblastoma cells growing at log phase (80% confluency) were trypsinized and seeded in multiwell plates at 4.5 x 10 ^6^ in triplicatesand incubated overnight to attach. The following morning, the media were replaced by media containing 20 μM Haldol or DMSO as vehicle control. The cells were counted every other day for 6 days using a hemocytometer or the Countess II (Invitrogen) cell counter.

**Immunoprecipitation (IP):**

To identify IQGAP1-interacting proteins in each condition, immune complexes from IQGAP1-specific antibodies and IgG controls were analyzed by mass spectrometry. The IP was prepared as previously (Osman et al., 2020) . Briefly, cells growing at ~ 80% confluency were treated with 20μM Haldol or DMSO as drug-vehicle control and incubated in cell culture incubator for 48 hrs. Total cell lysate was prepared by rinsing the cells with ice-cold PBS followed by scraping into ice-cold NP40 lysis buffer [20 mmol/L Tris (pH 8.0), 137 mmol/L NaCl, 1% NP40, 5% glycerol] supplemented with a cocktail of protease inhibitors (1 mmol/L phenylmethylsulfoxide, 10 mg/mL aprotinin, 10 mg/mL leupeptin) and 3 mmol/L Na3VO4. The lysates were cleared by centrifugation at 4 ^o^ C and protein concentration was determined by bicinchoninic acid assay (Pierce, Rockford, IL). Four hundreds to 1 mL of lysates were pre-cleared with 15 μl of NP40-equilibrated 50% slurry of protein A beads for 1 hr. and used for the immunoprecipitation (IP) reaction. 5 μL primary IQGAP1-specific antibody or IgG as control were added to the cleared cell lysates. The samples were rotated back-to-back for three hours at 4 ^o^ C followed by the addition of 15 μl agarose bead slurry and the samples were rotated for another three hours and centrifuged at 10,000 rpm for one minute. The supernatant was aspirated and the pellet was washed 3X by adding 1 mL of NP-40 and repeating the centrifugation, and 40 μl of the buffer was added to resuspend the beads. The samples were heated at 100°C for seven minutes and incubated on ice for three minutes. The samples were centrifuged for two minutes, resolved by SDS-PAGE and the gels were Coomassie stained.

**Mass Spectrometry** :

Mass spectrometric peptide analyses was performed using the Coomassie stained in-gel digest with trypsin per Bruker Daltonik instruction manual that was modified from Shevchenko et al, 1996 protocol. Briefly, the gel lanes were carefully cut into 1mm ^2^ pieces via clean scalpel blades and individually placed in Eppendorf tubes and covered with 25 mM NH _4_ HCO _3_ . Samples were vortexed for ten minutes and the supernatant discarded. 25 μl of 10 mM Dithiothreitol was added to each tube, and the samples were vortexed for five minutes followed by supernatant removal, and an hour resting period. Thereafter, 25 μl of 55 mM Iodoacetamide was added, the samples were vortexed, the supernatant removed, and the samples left to rest for 45 minutes. The gels were then dehydrated with 100 μl of 25 mM NH _4_ HCO _3_ in 50% Acetonitrile (ACN), vortexed for five minutes, and rested for 20 minutes. Trypsin (5-25 μl) was added to cover the gel pieces and the samples placed on ice, spun, and incubated overnight. Finally, 1 μl of each sample was loaded onto the MALDI-TOF (Bruker Ultraflextreme) and the corresponding mass to charge (m/z) ratios, spectra, and intensity values were recorded and used for data analyses.

**Data Analyses:**

For sample identification from mass-spectrometry, each sample MS-Spectrum was compared to known protein mass-spectra using the Mascot Search Engine. Sample analyses were done using the free online Mascot Program within the Protein Pilot Software (Version 5.0.2). The UniProt (2022_05 release) and the NIH-NCBI Blast Program were used to identify and describe the corresponding proteins from each sample.

**Statistical Analyses:**

Data are representative of at least three independent experiments with 2-3 replicas each. Statistical analyses were performed using Graph Pad Prism 6.0 (Graph Pad Software, Inc., La Jolla, CA, USA) and the algorithms in Microsoft Excel software (Version 2018) were also used to compare levels between different groups. All statistical tests (t-test and ANOVA) were two-sided, and *p* values less than 0.05 were considered statistically significant.

**Biosafety:**

All experiments were done following the outlined safety measures of each product and the University of Toledo's biosafety guidelines. All laboratory equipment were sanitized, calibrated and used for their intended purpose. Chemicals were used in a ventilated chemical fume hood.

## Reagents

**Table:** 

**Cell Lines**	**Origin**	**Description**	**Source**
MDA-MB-231 (HTB-26)	Triple negative breast cancer cell line isolated from a 51 years old Caucasian patient	Isolated from a pleural effusion. Primary adenocarcinoma, tumorigenic, and metastatic.	ATCC
MDA-MB-468 (HTB-132)	Breast cancer cell line isolated from 40 years old African American female		
MCF7 (HTB-22)	Breast cancer cell line isolated from a 69 years old Caucasian female		

**Table:** 

**Molecular Tools**	**Function**	**Description/Specification**	**Source**
Acrylamide	Assists in creating gel matrix to separate polypeptides using gel-electrophoresis	Acrylamide Solution (99.9% purity)	Bio-Rad
Ammonium Persulfate	Catalyst for acrylamide polymerization	Oxidizing agent	
Anti-IQGAP1 Antibody (Primary Antibody)	Monoclonal Antibody	Protein derived from the C-terminal half of IQGAP1	Thermo-Fisher
Dithiothreitol (DTT)	Disrupt polypeptide disulfide bonds	C₄H₁₀O₂S₂	
Haloperidol	FDA-approved Antipsychotic drug, Antagonist of D2, D3, and D4 dopamine receptor	C _21_ H _23_ ClFNO _2_ Induces apoptosis of neurons in the rat’s striatum.	Fisher-Scientific Sigma
HyClone Dulbecco’s Modified Eagle Medium (DMEM)	Basal medium used to sustain cell culture growth	High Glucose medium supplemented with 10% Fetal Bovine Serum (FBS), A/A (antibiotic and antifungal).	
Iodoacetamide (IAA)	Alkylating reagent to assist in protein characterization and mapping	C _2_ H _4_ INO	Thermo-Fisher
Lysis Buffer	Assist in breaking-opening cells for total protein isolation	Buffer (20 mL) supplemented with two phosphatase inhibitor tablets and 200 μl Protease Inhibitor Solution.	
1% NP-40 Cell Lysis Buffer	Serve as a wash buffer to assist in impurity removal experimental samples and for isolating proteins	10 mL NP-40 20 mL IM Tris, pH 8.0 8 g NaCI 100 mL glycerol 0.184 g Sodium Vanadate 1L ddH _2_ O	
Precision Plus Protein (SDS-Page Protein Standard)	A cocktail of known proteins used as a ruler to identify the sample	Mixture of 10 recombinant proteins of varying weights (10-250 kD).	Bio-Rad
Tetramethyl- ethylenediamine (TEMED)	Catalyst for acrylamide polymerization	Used together with ammonium persulfate to prepare acrylamide gel.	
Tris Resolving Gel Buffer	Helps to cast the resolving portion of the SDS-PAGE	1.5 M Tris-HCl, pH 8.8	
Tris-buffered saline Tween (TBST)	Wash buffer	Supplemented with 0.1% Tween 20 detergent	Thermo-Fisher
Trypsin	An enzyme used to digest proteins into peptides to facilitate protein analyses prior to running mass spectrometric analyses	Manufactured to resist autolytic digestion. Undergoes TPCK treatment to provide specificity. Purified by affinity chromatography prior to shipping.	Promega
